# Associations between plasma lysophospholipids concentrations, chronic kidney disease and the type of renal replacement therapy

**DOI:** 10.1186/s12944-019-1040-5

**Published:** 2019-04-04

**Authors:** Anna Michalczyk, Barbara Dołęgowska, Rafał Heryć, Dariusz Chlubek, Krzysztof Safranow

**Affiliations:** 1 Department of Biochemistry and Medical Chemistry, Pomeranian Medical University in Szczecin, Al. Powstańców Wielkopolskich 72, 70-111 Szczecin, Poland; 2 Department of Psychiatry, Pomeranian Medical University in Szczecin, ul Broniewskiego 26, 71-460 Szczecin, Poland; 3 Department of Microbiology, Immunology and Laboratory Medicine, Pomeranian Medical University in Szczecin, Al. Powstańców Wielkopolskich 72, 70-111 Szczecin, Poland; 4 Department of Nephrology, Transplantology and Internal Medicine, Pomeranian Medical University in Szczecin, Al. Powstańców Wielkopolskich 72, 70-111 Szczecin, Poland

**Keywords:** Lysophosphatidic acid, LPA, Lysophosphatidylcholine, LPC, Lysophospholipids, Bioactive lipids, Diagnostic marker, Chronic kidney disease, Renal failure, Renal replacement therapy

## Abstract

**Background:**

Lysophosphatidic acid (LPA) and lysophosphatidylcholine (LPC) are bioactive lysophospholipids involved in the pathogenesis of renal diseases, especially the renal fibrosis. Plasma LPC concentrations in chronic kidney disease (CKD) patients are lower or similar to those observed in control groups, but less is known about the LPA concentrations. The main aim of the study was the analysis of associations of chronic kidney disease and renal replacement therapy with the plasma LPA concentrations. We have also analyzed the relationship between the plasma concentrations of LPA and LPC.

**Material and methods:**

Study group consisted of 110 patients with CKD in stages G3-G5 according to the KDIGO guidelines and was divided into four subgroups: treated conservatively (CT, 30 patients), on hemodialysis (HD, 30 patients), on peritoneal dialysis (PD, 30 patients) and renal transplant recipients (RT, 20 patients). In HD the blood was collected immediately before (HD D1) and after the dialysis (HD D2). In RT the blood was collected immediately before (RT D1) and 3–14 days after the transplantation (RT D2). The control group (Con) consisted of 50 healthy volunteers. Plasma concentrations of LPA and LPC were measured using enzyme-linked immunosorbent assays.

**Results:**

In CT, PD and RT D2 plasma concentrations of LPA were significantly higher, compared to Con. In HD, LPA levels did not differ compared to Con and they were significantly lower compared to PD (HD D1 and HD D2), RT D2 (HD D1 and HD D2) and CT (HD D1). However, in most of patients concentrations of LPA were within the range of reference values established in healthy volunteers. Concentrations of LPC were significantly lower in almost all patients subgroups, compared to Con, except in PD. There were no significant correlations between plasma concentrations of LPA and LPC in any of patients subgroups.

**Conclusions:**

Presence of CKD is associated with increased plasma LPA levels and the hemodialysis therapy reduces this influence. However, only in a small percentage of patients with CKD, LPA concentrations are out of the reference range, which makes LPA not useful as a diagnostic marker for CKD. Further studies are needed to confirm and explain observed relationships.

## Background

Chronic kidney disease (CKD) is important problem of public health. As shown in the Global Burden of Disease research (GBD), the mortality caused by CKD increased in the world by over 30% between 2005 and 2015, reaching over 1.2 million deaths in 2015. Frequency of CKD in adults in the world is estimated at approx. 11–13%. [1]. According to the results of the European Renal Association -European Dialysis and Transplant Association (ERA-EDTA) reports, the incidence of end-stage chronic kidney disease (ESRD) increased in Europe in 2010–2014 despite the decline in the age-adjusted ESRD observed in many European countries. Probably this observation is caused by the increase in the percentage of elderly people in the population [2, 3].

CKD is associated with variety abnormalities in biochemical parameters of blood, including abnormalities in lipidomic profile. We decided to verify if this dysfunctions includes the changes in the levels of bioactive lysophospholipids in plasma. Lysophospholipids are involved in many physiological and pathological processes. The biological activity of lysophospholipids is based on the stimulation of receptors present on the surface or inside the target cells. Among the lysophospholipids, lysophosphatidic acid (LPA) and lysophosphatidylcholine (LPC) seems to be most involved in the pathogenesis of renal diseases.

Plasma levels of LPC are dependent on activity of lecithin:cholesterol acyltransferase (LCAT). In CKD patients, activity of LCAT is decreased, which is probably caused by decreased liver synthesis of this enzyme [4–8] and the plasma concentrations of LPC are lower [9, 10] or similar [11] to those observed in control groups. Less is known about the plasma LPA concentrations in CKD patients.

Previous studies on animal and human renal mesangial cells show that LPA stimulates proliferation, induces contractility, inhibits apoptosis of the cells and induces production of connective tissue growth factor (CTGF) [12–14]. Studies on animal proximal tubular cells showed that LPA stimulates proliferation, inhibits apoptosis of these cells and increases secretion of cytokines CTGF and platelet-derived growth factor B (PDGF-B) [13, 15]. LPA may also increase the expression and secretion of CTGF in human renal fibroblasts [16].

In vivo studies show that procedure of unilateral ureteral obstruction (UUO) in mice in order to initiate processes of interstitial tubular fibrosis, results in 3 fold increase in levels of LPA released from renal explants for several days after the procedure compared to the baseline levels. Changes in expressions of LPA receptors (↑LPA1, ↓LPA3) were also observed. Application of LPA1 receptor antagonist (Ki16425) significantly inhibits the fibrosis and decreases expression of CTGF and TGF-β [17]. Usage of LPA1 selective antagonist AM095 also resulted in decreased intensity of processes of fibrosis [18]. Similar effects were observed in LPA1 knock-out mice [17]. Studies on the mice model of nephrotoxic serum (NTS) nephritis, characterized by rapidly progressing inflammation with slow development of tubulo-interstitial fibrosis and as a result - the development of progressive renal failure showed almost 6-fold increase in expression of LPA1 receptor in this animals [13]. Above studies indicate the role of LPA and its receptors in renal fibrosis.

Interestingly, LPA seems to be protective factor against the renal ischemia-reperfusion injury (I/R) which is important cause of acute renal failure in patients after renal transplantation, major surgical procedures or in hypovolemic shock [19, 20].

Single reports showed the presence of higher LPA concentrations in blood [9] or urine [21] of patients with kidney diseases. Thus, the main aim of the study was the analysis of associations of chronic kidney disease and renal replacement therapy with the plasma lysophosphatidic acid concentrations. We have also decided to analyze the LPC plasma concentrations to confirm previous observations and analyze the relationship between the plasma concentrations of LPA and LPC.

## Material and methods

Study group (CKD) consisted of 110 patients of Department of Nephrology, Transplantology and Internal Medicine, Pomeranian Medical University in Szczecin, Poland with chronic kidney disease in stages G3-G5 according to the KDIGO guidelines [22]. The study group was divided into four subgroups: treated conservatively (CT, 30 patients), on hemodialysis (HD, 30 patients), on peritoneal dialysis (PD, 30 patients) and renal transplant recipients (RT, 20 patients). The inclusion criteria were: a) documented chronic kidney disease at stages G3-G5, b) age ≥ 18 years old, c) in subgroups of patients on dialysis, regular hemodialysis or peritoneal dialysis for at least 3 months before beginning of the study. The exclusion criteria were: a) the lack of informed consent, b) pregnancy, c) acute inflammatory diseases and d) diagnosed cancer.

The control group (Con) consisted of 50 healthy volunteers matched by age and sex, all of Polish descent. The inclusion criteria in this group were age ≥ 18 years old and the lack of renal diseases in anamnesis. The exclusion criteria were the same as in the CKD group, and additionally estimated glomerular filtration rate (GFR) below 90 ml/min/1.73 m^2^. Characteristic of the study participants is shown in Table 1.

**Table 1:** General characteristics of the study group and its subgroups and the control group

Group/ Subgroup	Group size (*n*)	Gender		Age [years]		BMI [kg/m^2^]	
		W	M	mean ± SD	Median	mean ± SD	median
CKD	110	55	55	54.0 ± 15.1	56.5	25.9 ± 4.0	25.4
CT	30	15	15	54.5 ± 16.1	56.5	27.6 ± 5.1	26.8
HD	30	14	16	53.8 ± 16.7	58.5	24.6 ± 3.9	24.6
PD	30	14	16	52.9 ± 15.5	54.0	25.5 ± 2.7	25.6
RT	20	12	8	55.0 ± 11.1	59.0	26.1 ± 3.8	25.1
Con	50	24	26	52.2 ± 9.3	55.0	25.7 ± 3.3	25.7
Statistical significance (p)		0.89*		0.61**		0.19**	

* p value for differences between CT, HD, PD, RT and Con in Chi-squared test; ** p value for differences between CT, HD, PD, RT and Con in Kruskal-Wallis test;

*CKD –patients with chronic kidney disease, CT –patients treated conservatively, HD –patients on hemodialysis, PD –patients on peritoneal dialysis, RT– renal transplant recipients, Con – control group, W – women, M – men, SD – standard deviation*

The main causes of the disease in CKD patients were glomerulonephritis (23%), diabetes mellitus (19%) and arterial hypertension (18%). According to the KDIGO guidelines in the subgroup of CT, there were 12 patients (40%) with CKD in stage G3, 11 (37%) in G4 and 7 (23%) in G5. In subgroups of patients on dialysis (HD, PD) the mean time of dialysis did not differ significantly (median 23 months for HD and 25 months for PD, *p* = 0,89). In HD subgroup hemodialysis sessions took place regularly three times a week (12 h per week) using polysulfone dialysis membranes. Among the patients on peritoneal dialysis, there were 23 patients on automatic peritoneal dialysis (ADO), and 7 on continuous ambulatory peritoneal dialysis (CADO). During peritoneal dialysis, standard dialysis concentrate containing glucose was used. In the subgroup of kidney transplant recipients, the organs came from deceased donors and the patients were treated with variety of immunosuppressive drugs. 75% of patients had previously undergone renal replacement therapy (HD and / or PD), 5% were not previously dialyzed, in the remaining 20% of patients there were no data on previous dialysis. In two patients, it was the second transplant due to failure of the transplanted kidney. In 3 patients delayed graft function (DGF) occurred.

The study was performed using the plasma of peripheral blood collected on K_2_EDTA. In subgroups of CT and PD, blood was collected from the peripheral vein during the follow-up visit. In hemodialyzed patients, blood was collected from the arteriovenous fistula immediately before (HD D1) and immediately after the hemodialysis (HD D2). In kidney transplant recipients, blood was collected from the peripheral vein before (RT D1) and 3–14 days after transplantation (RT D2). In patients from the Con and CT subgroup, blood was collected in the fasting state. In HD, PD and RT subgroups patients were not always fasting during the collection, due to different collection times and/or long duration of dialysis. Blood samples were centrifuged (1000 g, 10 min, 20 °C) within about 30 min of collection. Supernatant was transferred to new tubes and obtained plasma samples were stored at − 80 °C until the assays were performed.

Plasma levels of LPA and LPC were measured with the ELISA technic using Lysophosphatidic Acid Assay Kit II (K-2800S; Echelon Biosciences Inc., USA) and Enzyme-linked Immunosorbent Assay Kit For Lysophosphatidylcholine (CEK621Ge; Cloud-Clone Corp., USA). Absorbances in the samples were measured using plate reader EnVision 2104 Multilabel Reader (Perkin Elmer) and the standard curves were fitted using the program Wallac EnVision Manager 12.1.

The statistical analysis of the results was carried out using the Statistica 12 program (StatSoft Inc.). The Shapiro-Wilk test was used to analyze the normality of distributions. For the tested parameters, the distribution significantly deviated from the normal one, therefore non-parametric tests were used to analyze the results. The analysis of differences between groups was performed using the Mann-Whitney U test for comparisons between two groups and the Kruskal-Wallis test for comparisons between more groups. To evaluate changes in the concentration of a given parameter over time, the Wilcoxon signed-rank test was used. For analysis of correlations, Spearman’s rank correlation coefficient (R_s_) was used. Differences between groups were considered as statistically significant at *p* < 0.05.

## Results

In most of the CKD patients subgroups, mean plasma concentrations of LPA were higher and LPC lower, compared to controls (Table 2).

**Table 2:** Concentrations of plasma lysophospholipids in the subgroups of chronic kidney disease patients and the control group

Parameter		Subgroup							
		CT	HD		PD	RT		Con	P*
			D1	D2		D1	D2		
LPA [μM]	Mean ± SD	1.61 ± 1.35	0.99 ± 0.78	1.41 ± 1.75	2.43 ± 1.99	1.31 ± 1.02	2.12 ± 1.33	0.96 ± 0.58	< 0.001
	Median	1.28	0.94	0.80	1.96	0.89	1.74	0.82	
LPC [μM]	Mean ± SD	6.29 ±3.24	5.69 ±3,34	6.74 ±3.89	7.52 ±3.37	7.01 ±3.87	7.00 ±3.48	9.06 ±4.02	< 0.001
	Median	5.61	5.12	5.82	6.46	5.98	6.65	8.51	

*p value for differences between CT, HD D1, HD D2, PD, RT D1, RT D2 and Con in Kruskal-Wallis test;

*LPA – lysophosphatidic acid, LPC – lysophosphatidylcholine, CT –patients treated conservatively, HD D1–patients on hemodialysis before the dialysis (1st donation), HD D1–patients on hemodialysis after the dialysis (2nd donation), PD –patients on peritoneal dialysis, RT D1– renal transplant recipients before the transplantation (1st donation), RT D2– renal transplant recipients after the transplantation (2nd donation), Con – control group, W – women, M – men, SD – standard deviation*

**Fig. 1:**
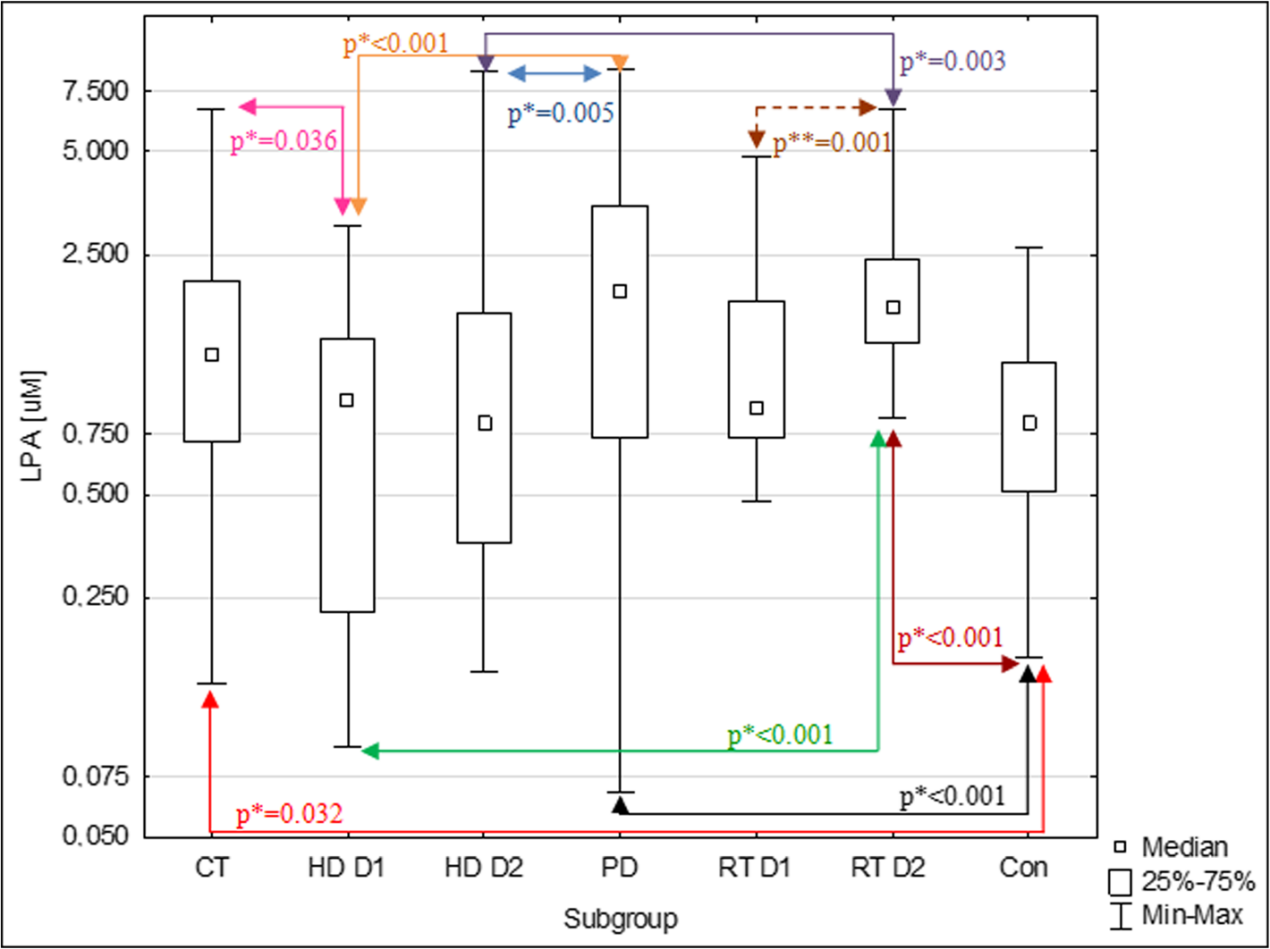
Plasma LPA concentrations in subgroups of CKD patients and the control group. Levels of LPA are presented in logarithmic scale. * p value for differences between groups in Mann-Whitney U test ** p value for differences between measurements D1 and D2 in Wilcoxon signed-rank test LPA – lysophosphatidic acid, CT –patients treated conservatively, HD D1–patients on hemodialysis before the dialysis (1st donation), HD D1–patients on hemodialysis after the dialysis (2nd donation), PD –patients on peritoneal dialysis, RT D1– renal transplant recipients before the transplantation (1st donation), RT D2– renal transplant recipients after the transplantation (2nd donation), Con – control group

In the CT, PD and RT D2 plasma concentrations of LPA were significantly higher than in Con. In the HD D1 and D2 LPA levels did not differ compared to Con and they were significantly lower compared to PD and RT D2. Concentrations of LPA in samples from HD D1 were significantly lower compared to CT. LPA levels in samples from RT D1 did not differ significantly compared to any other studied groups (Fig. 1).

In our previously published studies we established the range of plasma LPA reference values on the basis of material from 100 healthy volunteers (separate for women and for men, because our studies showed that the levels of LPA are significantly higher in women) [23]. In the current study we decided to check if the levels of LPA observed in CKD patients go beyond this range. As shown in Table 3, despite described above significant higher LPA levels in patients compared to Con, in all subgroups of patients at least half of observed concentrations was in the reference range.

**Table 3:** Classification of plasma levels of LPA in CKD patients according to reference values established in our previous study [23]. The table shows the number and percentage of patients in whom LPA level was below, within or above this range

Parameter	Classification	Subgroup					
		CT	HD		PD	RT	
			D1	D2		D1	D2
LPA*	Above reference range	7 23%	4 13%	6 20%	14 47%	4 13%	6 20%
	Within reference range	22 73%	23 77%	23 77%	15 50%	26 87%	24 80%
	Below reference range	1 3%	3 10%	1 3%	1 3%	0 0%	0 0%

*LPA reference range between 2,5–97,5 percentile (0,18–2,61 μM for women and 0,17–1,88 μM *for men)* [23]

**Fig. 2:**
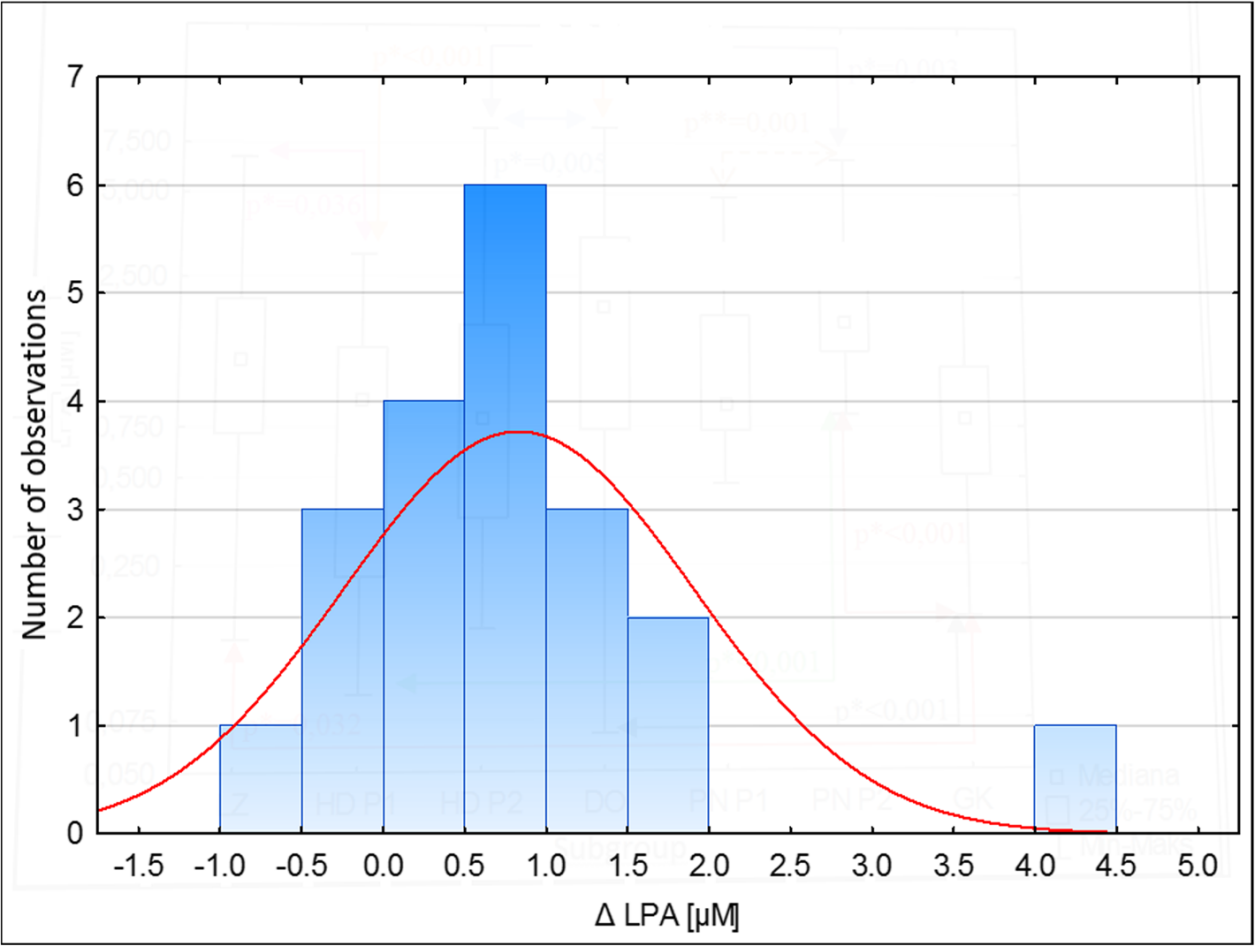
Histogram presenting changes (Δ) in plasma LPA concentrations after the renal transplantation compared to the levels before the procedure in renal transplant recipients (RT). Positive values indicate an increase, and the negative – a decrease in LPA levels

In subgroup HD, analysis of differences in LPA levels between donations (D1 and D2) did not show statistically significant changes in plasma LPA concentrations after the dialysis compared to the levels before the procedure (median ΔHD = + 0.14, *p* = 0,082). In most of the RT patients the plasma levels of LPA increased significantly after the renal transplantation compared to the levels observed before the surgery (median ΔRT = + 0.74, p = 0,001, Fig. 2).

**Fig. 3:**
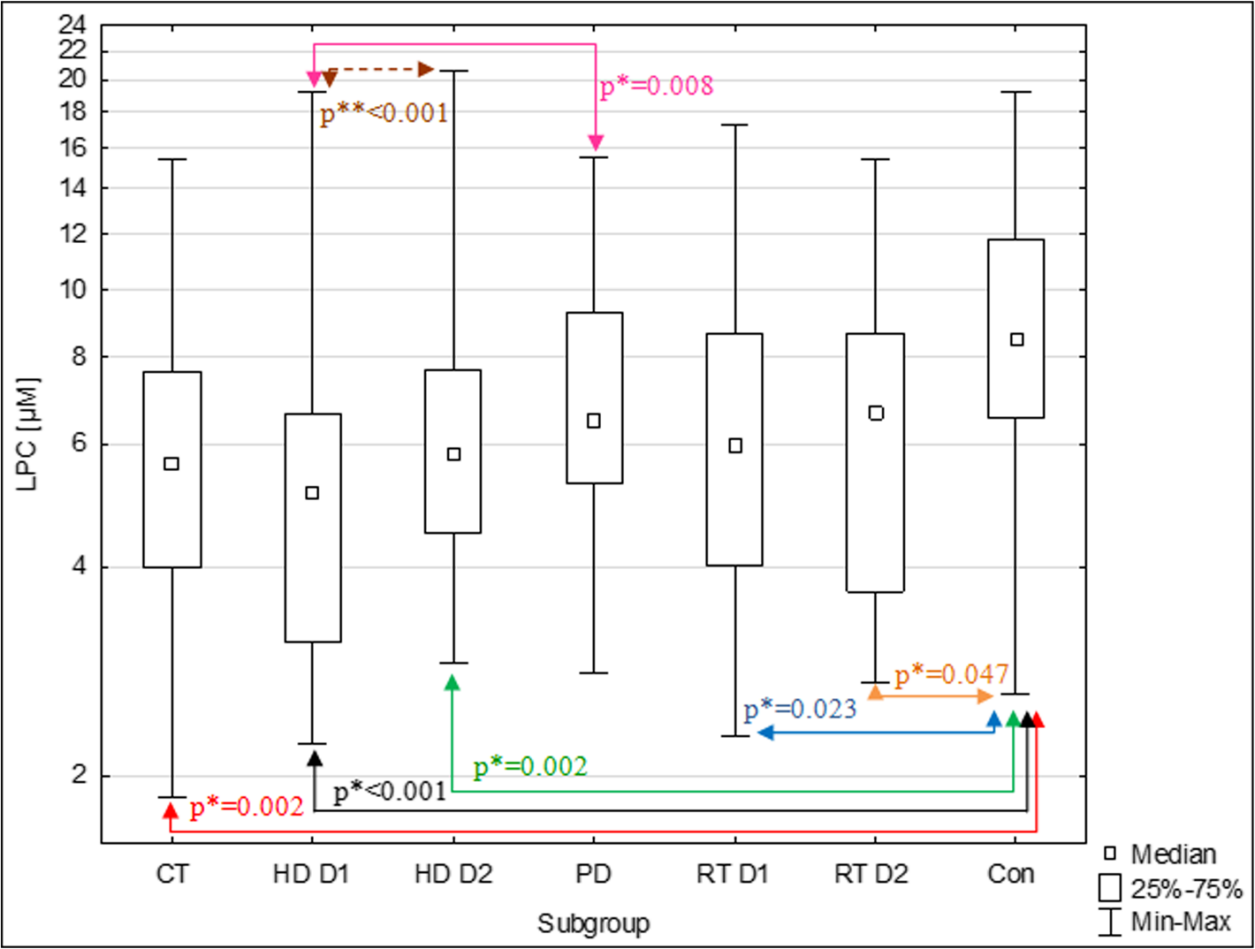
Plasma LPC concentrations in subgroups of CKD patients and the control group. Levels of LPC are presented in logarithmic scale. * p value for differences between groups in Mann-Whitney U test ** p value for differences between measurements D1 and D2 in Wilcoxon signed-rank test LPC – lysophosphatidylcholine, CT –patients treated conservatively, HD D1–patients on hemodialysis before the dialysis (1st donation), HD D1–patients on hemodialysis after the dialysis (2nd donation), PD –patients on peritoneal dialysis, RT D1– renal transplant recipients before the transplantation (1st donation), RT D2– renal transplant recipients after the transplantation (2nd donation), Con – control group

Analysis of LPC showed, that in CT, HD D1, HD D2, RT D1 and RT D2, LPC concentrations were statistically lower compared to Con (Fig. 3). In the PD, median LPA levels were also lower than in Con, but the differences did not reach the limit of statistical significance (*p* = 0.064). The only statistically significant difference observed between particular patients subgroups, was lower level of LPC in HD D2 compared to PD.

In HD, the dialysis was associated with significant increase of LPC levels (median ΔHD = + 0,79, *p* < 0.001). In RT, renal transplantation did not cause any significant changes in LPC concentrations (median ΔRT = + 0.01, *p* = 0.94).

Analysis of correlations between LPA and LPC did not show significant associations in any of the studied subgroups.

## Discussion

Considering the influence of LPA on CKD progression, we decided to check if renal failure is associated with increased plasma levels of LPA. In subgroups CT, PD and RT D2, compared to Con, significantly higher levels of LPA were observed. However, despite this observation, small percentages of patients with the concentration of LPA above the reference range indicate, that LPA is not useful as a marker for kidney damage, and based on the measurement of LPA concentration in most patients with CKD, it is not possible to distinguish them from healthy people. However, this does not change the fact, that the above subgroups of patients are exposed to statistically significantly higher concentrations of LPA in plasma than healthy individuals. Taking into account the complex and not fully known influence of LPA on human body, this increased exposition may have health consequences, that are difficult to predict.

Lack of significant differences in LPA levels between CT and PD subgroups indicates, that there is no significant influence of peritoneal dialysis on plasma LPA concentration. We did not find any previous studies on plasma LPA levels in peritoneal dialyzed patients or renal transplant recipients, which makes it impossible for us to compare the results of our studies with literature data. In the study concentrated on diabetic nephropathy, Saulnier-Blache et al. [21] did not find any significant differences in plasma LPA levels between diabetic patients with and without diabetic nephropathy (defined as an elevated urinary albumin / creatinine index with or without reduction eGFR < 60 ml / min / 1.73 m^2^). Differences between our and the cited study may result from a different profile of studied groups. In our study most of the patients were in CKD stages G4–5, while in cited study all patients were in stages G1-G3 (with albuminuria) according to KDIGO guidelines. The control groups also differed (healthy people with GFR ≥ 90 ml/min/1.73 m^2^ vs diabetic patients, GFR ≥ 60 ml/min/1.73 m^2^), which could affect the final result.

In our study in HD patients, levels of LPA both before and after the dialysis did not differ from Con. Significant differences in LPA levels between HD (D1 and D2) and PD patients indicate a significant influence of the type of dialysis therapy on plasma LPA concentrations. A single hemodialysis procedure did not cause significant changes in LPA levels, so there is probably no loss of LPA throughout the dialysis membrane. One of the hypothetical explanations for relatively low plasma concentrations of LPA in hemodialyzed patients could be the use of heparin. It was shown that heparin has inhibitory effect on phospholipase A2, the activity of which increases in patients with CKD and is a potential source of plasma LPA [24]. These results indicate a different effect of hemodialysis, compared to other treatments, on plasma LPA in patients with CKD. There is only one previous report on plasma LPA in hemodialyzed patients [9]. In this study, significantly higher concentrations of LPA were found in hemodialyzed patients prior to dialysis (1.41 ± 0.68 μM) than in the control group (0.54 ± 0.31 μM). However, this study was conducted on a small study groups (18 patients and 15 control subjects) and the mean age differed between groups by about 10 years, which could have an impact on the results because, as shown in our previous study, plasma LPA levels seem to be positively correlated with age [23]. Our study also differed from the cited report as regards the mean time of dialysis therapy (29 ± 22 months vs 123 ± 85) and used method of LPA levels measurement (ELISA vs gas chromatography). The source of the discrepancies could also be difference in the methods of hemodialysis (not described in the cited work), resulting from the fact that both studies are divided by almost 20 years. The type of used dialysis membrane and its biocompatibility may significantly affect LPA levels, as they may trigger platelet activation to a different extent [25, 26].

The lack of significant differences in LPA levels in the RT subgroup before transplantation in relation to any of the other subgroups of patients and the control group, may results from the fact that the subgroup of PN consisted of both hemodialyzed, peritoneal dialyzed and conservatively treated patients, with the majority of patients being hemodialyzed. In this subgroup after transplantation, there was a significant increase in LPA concentration to a level not significantly deviating from the concentrations observed in the LZ and DO subgroups. Due to the relatively short time from kidney transplantation, it is difficult to say whether this change was related to discontinuation of hemodialysis, transplant function, surgery or the use of immunosuppressive drugs. An increase in plasma LPA after transplantation could also be a potentially beneficial protective mechanism against ischemia-reperfusion (I/R) injury that is a significant cause of acute renal failure in renal transplant recipients [19, 20].

Plasma concentrations of LPC were significantly lower in almost all patients groups, compared to Con. The results of our research are broadly consistent with those published so far. The Sasagawa et al. [9] found significantly lower LPC concentrations in the plasma of CKD patients on hemodialysis compared to the control group (115 ± 30 μM, *N* = 18 and 133 ± 19 μM, *N* = 15respectively,). In the study of Lee et al. [10], the same relationship was found in serum (268 ± 65.9 μM, *N* = 69 and 332.7 ± 57.2 μM, *N* = 33). Vecino et al. [11] did not observe significant differences in LPC concentration in the plasma of CKD patients in the pre-dialysis period compared to the control group (109.7 ± 41.6 μM, *N* = 7 and 80.4 ± 16.8 μM, *N* = 9), although the studied groups were relatively small. It is worth to notice that LPC levels obtained in our study are much lower, compared to the cited studies. In published studies, the mean LPC concentrations in the control groups ranged from 80 to 364 μM [9–11, 27–30]. A satisfactory recovery of the standard included in the kit (LPC 16:0) added to the two plasma samples (91.5 and 104.4%), indicates that our low measured LPC levels, are not the result of the matrix effect. The assay instructions do not provide information on its specificity for different LPC molecular species. Perhaps, the antibody used in the test reacts only with part of the LPC molecules, that contain certain fatty acids. Decreased LPC concentrations in CKD patients probably result from decreased LCAT activity in plasma. The study of Gillett et al. on the patients with newly diagnosed renal failure showed a lower percentage of LPC among phospholipids in plasma, lower levels of cholesterol esters and lower plasma LCAT [7] activity compared to the control group and the negative correlations between LPC and LCAT and plasma urea concentration. Reduced LCAT activity in different groups of patients with CKD was also found in other studies [4–6]. As evidenced by Lee et al. [10] the presence of reduced LPC concentrations observed in patients may have clinical significance. Five-year observation of hemodialyzed patients with CKD showed that the low serum LPC concentrations were associated with a significant increase in the risk of cardiovascular disease. This effect may be related to reduced LCAT activity in plasma.

Hydrolysis of LPC and other lysophospholipids to LPA, catalyzed by autotaxin (ATX) and its lysophospholipase D activity, is considered as main source of LPA in plasma [31]. A probable explanation for the lack of correlation between the levels of LPA and LPC observed in our studies, may be the saturation of the enzyme with a substrate. According to literature data, the Michaelis constant (Km) for reaction of hydrolysis of LPC to LPA, catalyzed by ATX is about 100 μM [32, 33] and this value is similar to the mean plasma levels of LPC in patients with renal failure [9, 11]. In our study, the LPC levels in patients were much lower, but as mentioned above, most likely this results from the different specificity of the assay towards different LPC molecular species. Hasogaya et al. [34] in the study on healthy subjects, did not observe significant correlations between plasma levels of LPA and LPC in a univariate analysis, despite a strong correlation of plasma LPA with serum ATX activity. Another explanation for the lack of correlation may be important role of other lysophospholipids (e.g. lysophosphatidylserine, lysophosphatidylethanolamine, lysophosphatidylinositol) as ATX substrates in blood. In study of Block et al. [35] no significant correlations between ATX activity and LPA levels were found, and supplementation with eicosapentaenoic acid and docosahexaenoic acid did not contribute to qualitative changes of LPA in plasma, despite the presence of such changes in LPC (increase in the proportion of LPC containing supplemented fatty acids), which may suggest a potential contribution of other enzymes (e.g. PLA2) or extravascular sources of LPA in the blood. Observed in our studies higher LPA levels in the plasma of CKD patients, with a simultaneous decrease in LPC levels and a lack of correlation between these two parameters, may indicate a significant contribution of other than LPC substrates or other than ATX enzymes in plasma LPA synthesis, but they can also be associated with changes in the activity of the LPA-degrading enzymes or its extravascular origin.

## Conclusions

Previous studies showed potential role of LPA in the pathogenesis of kidney disease, in particular in the process of renal fibrosis. In our studies we show for the first time, that patients with CKD treated conservatively and peritoneally dialyzed and the renal transplant recipients have higher plasma LPA levels compared to control group. We have also observed the increase in LPA levels after renal transplantation. We also confirmed that renal failure is associated with lower LPC levels. On the basis of our study, we can conclude, that the presence of CKD is associated with increased plasma LPA levels and the hemodialysis therapy reduces this influence. However, only in a small percentage of patients with CKD, LPA concentrations are out of the reference range, which makes LPA not useful as a diagnostic marker for CKD. Further studies are needed to confirm and explain observed relationships.

## Abbreviations

ADO: Automatic peritoneal dialysis
ATX: Autotaxin
CADO: Continuous ambulatory peritoneal dialysis
CKD: Chronic kidney disease
Con: Control group
CT: Patients treated conservatively
CTGF: Connective tissue growth factor
DGF: Delayed graft function
HD D1: Patients on hemodialysis before the dialysis (1st donation)
HD D2: Patients on hemodialysis after the dialysis (2nd donation)
HD: Patients on hemodialysis
KDIGO: Kidney Disease Improving Global Outcomes
LCAT: Lecithin:cholesterol acyltransferase
LPA: Lysophosphatidic acid
LPC: Lysophosphatidylcholine
PD: Patients on peritoneal dialysis
RT D1: Renal transplant recipients before the transplantation (1st donation)
RT D2: Renal transplant recipients after the transplantation (2nd donation)
RT: Renal transplant recipients
UUO: Unilateral ureteral obstruction
